# HEALTHCARE ACCESSIBILITY AND UTILIZATION AMONG LGBTQ+ OLDER ADULTS

**DOI:** 10.1093/geroni/igac059.1950

**Published:** 2022-12-20

**Authors:** Jessika Dayrit, Ana Maria Pasatiempo, Shirley Salom-Bail, Cesz Islaya

**Affiliations:** Washington University in St. Louis, Menlo Park, California, United States; U.S. Department of Veterans Affairs, Anchorage, Alaska, United States; US Department of Veterans Affairs, Menlo Park, California, United States; US Department of Veterans Affairs, Menlo Park, California, United States

## Abstract

**Background:**

Studies show disparities in healthcare have exacerbated during the COVID-19 pandemic, especially among Lesbian, Gay, Bisexual, Transgender, and Queer + (LGBTQ+) older adults. The aim of this paper is to understand health care utilization and accessibility among LGBTQ+ older adults and to examine if social assets, such as income, education, and employment are associated with health care utilization and accessibility.

**Methods:**

Data from BRFSS 2020 was used. Study focused on LGBTQ+ and non-LGBTQ+, age 65 and older, comparing health care utilization and accessibility. Data was weighted for complex sampling design. Logistic regression was used to examine the odds of health care accessibility and health care utilization while controlling for socioeconomic status.

**Results:**

Total sample size of study was 14,453. 6.14% of participants identified as LGBTQ+, which 11% of them were unemployed (CI=0.08-0.17), 21% earned less than $15,000 annually (CI=0.15-0.28), and 35% did not graduate from high school (CI=0.27-0.44). Our analyses indicated LGBTQ+ are less likely to have primary health care provider than non-LGBTQ+ (OR=0.64; CI=0.48-0.84); they are less likely to have health care insurance than non-LGBTQ+ (OR=0.60; CI=0.39-0.93). We did not find association if LGBTQ+ are less likely to seek medical help, when needed, due to cost (OR=0.72; CI=0.53-1.02). Our findings suggest disparities in socioeconomic status, such as income, employment, and education have significant association with health care accessibility and health care utilization. Limitation of study includes recall bias due to self-report. Longitudinal and qualitative research regarding healthcare utilization and accessibility among older LGBTQ+ population need to be explored.

